# How to See a Larynx

**Published:** 1909-07-10

**Authors:** 


### The General Practitioner's Column.

[Contributions to this Column are invited, and if accepted will be paid for.]

### HOW TO SEE A LARYNX.

### PRACTICAL NOTES BY A "G.-P.-LARYNGOLOGIST."

One thing that in student days our teachers used often, and with obvious sincerity, to impress upon us, was that the mistakes of clinical beginners arise not so much from ignorance of such and such an indication, as by neglecting to look for it. This aphorism has certainly an application to laryngeal affections, since morbid lesions in this region, unlike much in general surgery, are not difficult of diagnosis because of the ease with which they are recognisable from text-book pictures. Inspection of two or three appropriate cases will enable any medical man to distinguish, say, an angio-fibroma of the vocal cord from a papilloma growing in a similar position—which, for practical purposes, is rather a refinement in diagnosis. But he must look at the larynx. And to do this he must be master of a certain technique only to be acquired by practice and perseverance.

The first difficulty lies in the management of reflected light, which every progressive practitioner should be familiar with. Until this is mastered, even the fauces cannot be seen properly, whilst the diagnosis of aural troubles, so common in all classes, becomes mere guess work. To keep light focussed on a particular spot under all the exigencies of physical examination is a mechanical accomplishment to be practised and learnt. For use in general practice the most suitable illumination is undoubtedly an incandescent light. It is brilliant, and whiter than that from any form of electrical lamp, except the costly and cumbrous Nernst. This quality of whiteness is important, as the dark red colour of the mucosa, often present in laryngeal disease, lights up badly with a yellowish illumination. Other points in favour of the incandescent are that at a pinch the bull's eye attachment may be dispensed with, and that it serves to warm the throat mirror. It is usual to have the lamp at the patient's left hand, and on a level with the forehead of the practitioner, who looks through the hole in the head mirror with his right eye.

Two or three thicknesses of soft crinkled paper, similar to that used for lamp-shades, make a good tongue-cloth. The tongue should be extruded to its utmost, and the tip held between the observer's thumb and first and second fingers—the third and fourth raising the upper lip out of the line of vision. Fairly often the tongue is too moist and slippery to be grasped firmly; if so, it should be cleaned with the tongue-cloth stretched between the two hands, and the patient told to keep it out while a fresh dry cloth is being obtained. A No. 5 throat mirror, its face previously warmed until the condensation disappears, and the temperature of its back tested against the surgeon's cheek, is now arched over the dorsum of the tongue and pressed firmly upwards and backwards against the base of the uvula, the shank being steadied against the corner of the mouth. If the patient shows signs of any qualms, these may be lessened by telling him in a decided manner to go on breathing quickly through the mouth. If, however, they continue, it is best to remove the mirror and use cocaine. One of Sir Frederick Treves' dicta is that a physical examination which causes pain is not worth much; it is true, at all events, of laryngoscopy. The intermittent glimpses obtained during the struggles of a "Channel crossing" inspection are useless for diagnosis.

With the following procedure cocaine may be employed without any risk and without ill-effect. A one-grain dried preparation of cocaine is freshly dissolved in ten minims of tap-water, since solutions of this drug do not keep longer than a week. The patient is given a basin to hold in his lap, and warned to spit out at once after each application. A small wisp of cotton wool, held in angled forceps, is now dipped in the cocaine and drawn gently over the arch of the soft palate. This is repeated twice at intervals of a minute, and after a further five minutes the fauces are pale and anæsthetic. In four cases out of five what will appear first in the mirror will be mainly the upper surface of the epiglottis; that is, as it were, the lid of the voice-box instead of its interior. The manœuvre which almost infallibly brings about this lifting of the lid is to tell the patient to phonate "ee" shrilly, and at the same moment to twist the mirror slightly about its horizontal diameter (keeping it firmly pressed back), so that the lower half of its circumference rises and comes forward a little. Sometimes the patient cannot phonate. Then one must secure a laugh by the time-honoured device of asking him to "give a graceful smile." It is important to remove the mirror before "gagging" occurs, and to wait three or four minutes before re-examining. In this way a reliable laryngeal inspection may be made, and the commoner lesions—pachydermia, singer's nodes, characteristic tuberculosis, functional aphonia—identified with certainty. It can be done, too, without discomfort or annoyance to the patient; indeed, on the contrary, with a very beneficial mental and moral effect. There is nothing which so inclines patients to carry out treatment exactly as the favourable impression produced by a workmanlike preliminary examination of the site of disease.

